# Emphysematous Pyelonephritis in a Transplant Kidney

**Published:** 2010-02-01

**Authors:** M. Salehipour, J. Roozbeh, A. R. Rasekhi, M. A. Afrasiabi, H. Rezaee, K. Izadpanah, S. A. Malek-Hosseini

**Affiliations:** *Shiraz Organ Transplantation Center, Shiraz University of Medical Sciences, Nemazi Hospital, Shiraz, Iran*

**Keywords:** Emphysematous pyelonephritis, renal transplantation, urinary tract infection

## Abstract

Emphysematous pyelonephritis (EPN) is a severe necrotizing infection of the kidney and its surrounding tissues. It is characterized by the production of gas within the kidney and perinephric structures. EPN often affects diabetic women but can also occur in nondiabetic patients who have ureteral obstruction and in immunocompromised patients. Herein, we report EPN in a 23-year-old woman who had a renal transplantation.

## INTRODUCTION:

Renal transplantation is the preferred treatment for patients with end-stage renal disease (ESRD). In recent years, with the introduction of new immunosuppressive drugs, graft and patient survival have improved, but urinary tract infection (UTI) is still a common complication that can cause morbidity and mortality in a renal transplant recipient. Emphysematous pyelonephritis (EPN) is a life-threatening, fulminant necrotizing infection of the renal parenchyma associated with gas formation within kidney, the collecting system and/or perinephric space. While the majority of patients with EPN have diabetes mellitus (DM), urinary tract obstruction and immunodeficiency are other risk factors. Herein, we report a case of EPN in a renal transplant recipient.

## CASE REPORT

A 23-year-old non-diabetic woman with ESRD received a living unrelated renal transplant. Two months after the transplantation, on account of reduced urine out-put and rise in serum creatinine (Cr) level, the patient was evaluated. On physical examination, she was toxic, febrile though hemodynamically stable. Abdominal examination revealed redness and severe tenderness in the right lower quadrant (RLQ).

Color Doppler ultrasonography of the allograft was consistent with severe renal artery stenosis and decreased perfusion in the transplant kidney. She underwent angiography and a stent was inserted in the renal artery. Two days later, she developed high grade fever (T = 40 °C), chills, nausea, vomiting, gross hematuria and RLQ pain. At the time of presentation, she was taking 175 mg cyclosporine and 1000 mg mycophenolyte mofetil (MMF) twice a day, and 50 mg/day prednisolone. The pertinent lab findings included a WBC count of 37.2×10^3^, a hemoglobin level of 6.6 g/dL, a Cr of 10.8 mg/dL, glucose of 93 mg/dL, and a platelet count of 409×10^3^/µL. Urinalysis revealed many red cells and WBC and urine culture was negative.

**Figure 1:**
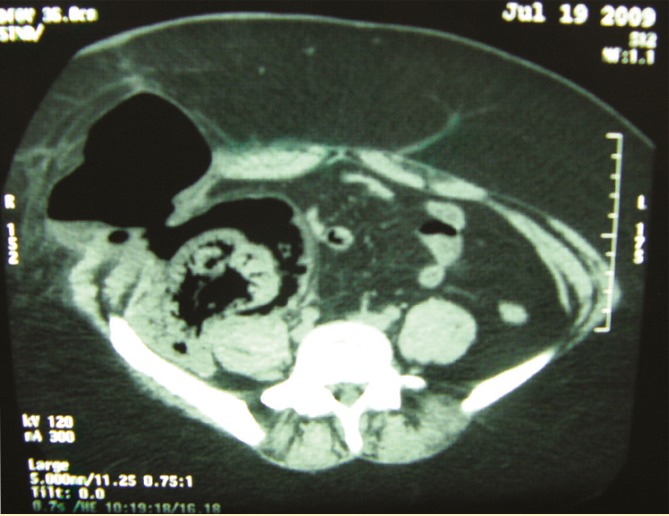
Non-contrast CT scan of the abdomen showing extensive extraperitoneal perinephric gas extending to paranephric space accompanied by renal parenchymal destruction

Sonography of the abdomen revealed small and atrophic native kidneys and a large air-fluid level in the right iliac fossa extending to the abdominal wall. The transplant kidney could not been delineated. A non-contrast CT of the abdomen demonstrated extensive retroperitoneal air around the transplant kidney which extended to subcutaneous tissues in RLQ (Fig. 1). 

The patient was treated with 250 mg intravenous imipenem q12h and 1 g intravenous vancomycin q96h, but she did not respond clinically and radiologically. Therefore, emergency allograft nephrectomy was performed. During the operation, about one liter of pussy, malodorous fluid was drained from the subcutaneous and peri-allograft space. The transplant kidney was very small, soft and dusky in appearance indicating the occlusion of renal artery.

Histopathologic examination of the transplant kidney showed infarcted kidney with severe acute and chronic inflammation of its pelvis and ureter.

The patient’s fever subsided soon after nephrectomy; she was discharged six days post-operatively in good condition.

## DISCUSSION

EPN is a fulminant, necrotizing infection of the renal parenchyma that is associated with gas formation in the kidney or in the perinephric space. Although the first case of EPN was reported by Kelly and Mac Cullem in 1898, however, Schultz and Klorfein first used the term EPN in 1962 [1, 2]. EPN should be differentiated from emphysematous pyelitis that is characterized by gas in the pelvicaliceal system only. Emphysematous pyelitis is a benign disease and a complete recovery can be achieved with medical treatment. By contrast, EPN has a high mortality.

EPN is usually seen in patients with DM with higher incidence in women (41:7, 3:1) [3, 4]. The left kidney is involved more frequently than the right kidney. Non-diabetic EPN patients show varying degrees of immunologic impairment such as tuberculosis, AIDS, alcoholism and renal transplantation [3, 6, 7].

*Escherichia coli* is the most common organism isolated in EPN but other Gram-negative organisms (*e.g.,* *Klebsiella, Proteus* and *Pseudomonas spp*), anaerobic organisms and fungi as well as cultures showing no growth have also been reported [5, 8, 9].

Diagnosis of EPN is made by radiological investigations; CT is the imaging modality of choice. It demonstrates presence, location and the extent of gas in the parenchyma and perinephric or paranephric space and also reveals renal destruction. According to CT findings, Huang and Tseng [3] have classified EPN into four classes as follows:

**Class 1:** Gas in the collecting system only.

**Class 2:** Parenchymal gas only.

**Class 3a**: Extension of gas into perinephric space.

**Class 3b:** Extension of gas into paranephric space.

**Class 4: **EPN presents in one or both kidneys.

Wan, *et al*, on the other hand, have classified EPN as types I and II based on CT findings.

Type I is characterized by renal necrosis together with the presence of gas but without fluid. Type II is characterized by parenchymal gas associated with fluid in the renal parenchyma, perinephric space or the collecting system. Our patient had features of class 3 and 4 of Huang and Tseng classification.

Optimal therapeutic modalities for EPN are controversial. There are various therapeutic approaches to EPN such as medical management alone, percutaneous drainage (PCD) under guidance of sonography or CT and nephrectomy. Medical management alone results in highest mortality [3, 5, 11].

Huang and Tseng showed that PCD and relief of the urinary tract obstruction (if present) combined with antibiotic therapy should be used in class 1 and 2 disease. However, they suggested that PCD combined with antibiotic therapy may be attempted for patients with extensive EPN (class 3 or 4) with less than two risk factors (*i.e.*, thrombocytopenia, acute renal failure, altered sensorium and shock); however, for those patients with two or more risk factors, nephrectomy can provide the best result [3]. Therefore, nephrectomy is limited to patients with the following criteria: a nonfunctional kidney, gross renal destruction, class 3 disease and existence of two or more risk factors.

Falagas and colleagues showed that conservative treatment alone, bilateral EPN, and type I EPN, were also associated with increased mortality. Therefore in the severe form of EPN or in very high risk patients (*e.g.*, renal transplantation), nephrectomy of the native kidney or the allograft is recommended and PCD should be reserved for early stage disease for the preservation of kidney function.
